# Fatal cases associated with pandemic influenza A (H1N1) reported in Greece.

**DOI:** 10.1371/currents.RRN1194

**Published:** 2010-11-09

**Authors:** Maria Athanasiou, Theodore Lytras, Georgia Spala, Eleni Triantafyllou, Kassiani Gkolfinopoulou, Georgios Theocharopoulos, Stavros Patrinos, Kostas Danis, Marios Detsis, Sotirios Tsiodras, Stefanos Bonovas, Takis Panagiotopoulos

**Affiliations:** ^*^MD, MPH, Department of Epidemiological Surveillance and Intervention, Hellenic Centre for Disease Control and Prevention, Athens, Greece; ^‡^MD, MPH, Department of Epidemiological Surveillance and Intervention, Hellenic Centre for Diseases Control and Prevention, Athens, Greece; ^§^Public Health Officer, Department of Epidemiological Surveillance and Intervention, Hellenic Centre for Disease Control and Prevention, Athens, Greece; ^¶^RN, MPH, PhD, Department of Epidemiological Surveillance and Intervention, Hellenic Centre for Diseases Control and Prevention, Athens, Greece; ^#^RN, MPH, Department of Epidemiological Surveillance and Intervention, Hellenic Centre for Disease Control and Prevention, Athens, Greece; ^**^Biostatistician, Department of Epidemiological Surveillance and Intervention, Hellenic Centre for Disease Control and Prevention; ^††^MD, Epidemiologist, Department of Epidemiological Surveillance and Intervention, Hellenic Centre for Disease Control and Prevention, Athens, Greece; ^§§^4th Department of Internal Medicine, Athens University Medical School; ^¶¶^Head, Department of Epidemiological Surveillance and Intervention, Hellenic Centre for Diseases Control and Prevention, Athens, Greece and ^##^Dept of child health, National School of Public Health, Athens, Greece; Dept of surveillance and intervention, Hellenic Centre for Disease Control and Prevention, Athens, Greece

## Abstract

ABSTRACT

Between 18 May 2009 and 3 May 2010, a total of 149 fatal cases associated with pandemic influenza A (H1N1) were reported in Greece. Detailed case-based epidemiological information was available for the large majority of fatal cases. The time distribution follows an epidemic curve with a peak in the beginning of December 2009 and a second peak one month later. This is similar to that of laboratory confirmed cases and influenza-like illness cases from our sentinel surveillance system, with two weeks delay. The most commonly reported underlying conditions were chronic cardiovascular disease and immunosuppression, while the most frequently identified risk factor was obesity. These findings should be taken into consideration, when vaccination strategies are employed.

## **INTRODUCTION**

Since the emergence of pandemic influenza A (H1N1) in April 2009 and the initial concerns about the risk of high mortality rates, much effort has been devoted to the understanding of the severity and impact of this novel virus.

By 10 June 2010, more than 18,138 deaths were associated with the pandemic worldwide [1], with approximately 2,900 deaths reported in Europe [2]. In Greece, the first laboratory confirmed case of pandemic influenza was detected on 18 May 2009. During summer 2009, influenza-like illness (ILI) rates remained low. The first fatal case associated with pandemic influenza A (H1N1) was reported on 23 August 2009; a young man with underlying cardiovascular disease.

This report describes the incidence of all fatal cases associated with pandemic influenza A (H1N1) in Greece. It sets out to highlight the factors associated with poor prognosis to inform the implementation of prevention and control programs. 

## **METHODS**

With the onset of the pandemic, an enhanced surveillance system for pandemic influenza A (H1N1) was set up, which included direct reporting to the Hellenic Centre for Diseases Control and Prevention (HCDCP) of each laboratory confirmed case of pandemic influenza A (H1N1) with a fatal outcome. The data collected for each case, using a standardized form, are listed in Table 1.

**Table 1. Data collected for every fatal case**

**Table:** 

Hospital
Demographics (age, sex, residence, nationality)
Laboratory
Time course of illness (date of onset, date of admission, date of death)
Underlying diseases
Complications
Treatment (antivirals, antibiotics)
ICU admission
Isolation
Intubation

Fatal cases were defined as those associated with pandemic influenza A (H1N1), providing that the infection was laboratory confirmed either before or after death, regardless of the underlying cause of death. With the onset of the pandemic, nasopharyngeal swab specimens collected from each case “under investigation” were sent to either of the two national reference laboratories. After 15 July 2009, when Greece moved to a mitigation phase, criteria for testing mainly included severe cases requiring hospitalization, and selected cases from clusters of influenza-like illness [3]. Due to the high number of specimens sent for testing (the total number of specimens was over 48,500), seven more laboratories were also introduced into the system [4]. The diagnosis was confirmed with real-time PCR.

Specimens were also sent for testing by physicians contributing to the sentinel surveillance system. They reported, on a weekly basis, the number of patients seen with ILI and the total number of visits.

During the pandemic, all cause mortality in Greece was also monitored as part of a European mortality monitoring system (EuroMoMo) [5]. The general objective of this system was to detect and measure, on a real-time basis, excess number of deaths from all causes.

## **RESULTS**

Between 18 May 2009 and 3 May 2010, a total of 149 fatal cases associated with pandemic influenza A (H1N1) were reported to the HCDCP. During this period, a number of 18,230 laboratory confirmed cases were recorded. With this denominator, the case fatality rate (CFR) was 0.82%. On a general population basis, this corresponds to 13.23 deaths per million.

Data from the EuroMoMo surveillance system during the same period did not show any significant increase in all-cause mortality both in the total population and in specific age-groups.

The time distribution of cases follows an epidemic curve with a peak in the beginning of December 2009 and a second peak one month later. It is similar to the time distribution of the sentinel surveillance system in place (ILI cases/1000 visits) with 2 weeks delay (Figure 1).

**Figure:**
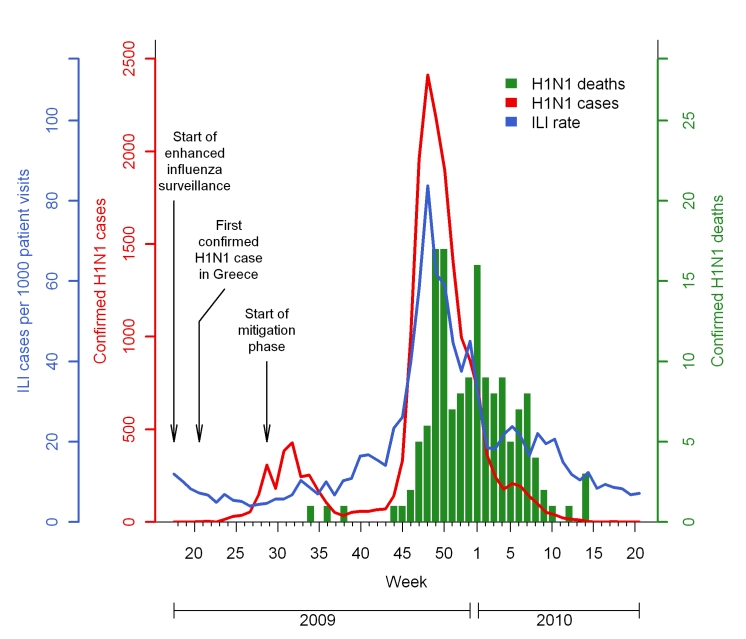


 **Figure 1: Time distribution of pandemic influenza A (H1N1) fatal cases, laboratory confirmed cases and ILI cases**

Data on sex, age and date of death were available for all fatal cases. Of the total 149 cases recorded, 85 were male (57.1%) and 64 were female (42.9%). Males appeared to have an increased risk of death (15.2 deaths per million) compared to females (11.3 deaths per million). However, the difference was marginally not significant (p=0.066).

The median age of the 149 fatal cases was 53 years (range 1–86). Eight of these (5.4%) were ≤ 18 years. The population mortality rate appeared to increase progressively with age. It was lower in young children aged 0–4 years (3.6 per million) and higher (20 per million) among those over 65 years old (Figure 2).



**Figure 2. Age distribution of pandemic influenza A (H1N1) fatal cases with population pandemic influenza mortality rate** 

Information on underlying disease was documented for 140 of 149 cases. Patients who died had a median of one long-term condition (28 had no long-term conditions, 52 had one, 35 had two, 18 had three, 5 had four, and 2 had five). Underlying disease was equally distributed between the sexes, but understandably not among age groups (p=0.001) (Figure 3). A high proportion of teenagers (57% of the 10–19 year-olds) and young adults (50% of the 20–39 year-olds) had no documented underlying disease, while all cases under the age of 10 years and over the age of 60 years had at least one underlying disease.

**Figure:**
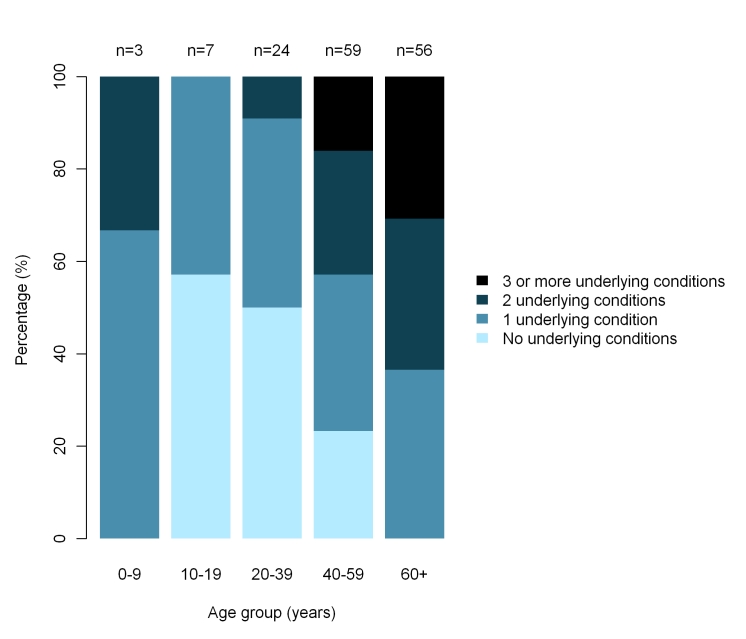


**Figure 3. Age distribution of pandemic influenza A (H1N1) fatal cases, by number of underlying conditions**

No fatal case occurred during pregnancy, and one death was recorded in a woman in the post-partum period (up to 28 days after delivery).Obesity, defined as a BMI ≥ 30kg/m^2^, was the most frequently identified risk factor. Thirty eight cases (26%) were obese.

Chronic cardiovascular disease and immunosuppression were the most commonly reported long-term conditions (Table 2). The median age of fatal cases was 69.5 years in those with a chronic cardiovascular disease and 54 years in those with immunosuppression. In children and adolescents ≤ 18 years old, the most commonly reported long-term condition was neurological disease (63%).

**Table 2. Distribution of underlying diseases for fatal cases**

**Table:** 

**Underlying disease**	**Total (%)^a^**
Chronic respiratory disease	35 (23.5)
Chronic heart disease	48 (32.2)
Immunosuppression	41 (27.5)
Metabolic disease	40 (26.8)
Chronic renal/liver disease	20 (13.4)
Chronic neurological disease	22 (14.8)

  ^a^ cases may suffer from more than one underlying diseases 

 History of alcoholism was reported in 11 fatal cases (7.4%), while history of psychiatric disorders (including depression) was reported in 8 cases (5.4%).

In cases for whom the date of symptom onset was stated (n=137), death occurred a median of 16 days after influenza-like symptoms began. Median time from onset of symptoms to hospital admission was 3 days (range 0–21). Ten cases (6.7%) developed influenza-like symptoms while already in hospital. The median length of hospitalization among all fatal cases was 13 days.

The majority of cases with a fatal outcome (86%) was treated in an intensive care unit (ICU), dying a median of 13 days after admission to the unit. The median age of those admitted to an ICU was 52 years. Of cases admitted to an ICU, 110 (89.4%) required assistance with ventilation. Patients admitted to an ICU were more likely to have a cardiological disorder (p=0.022), acute respiratory distress syndrome (ARDS) (p=0.015), or multiple organ failure (p=0.008) than patients not admitted to an ICU. In addition, cases treated in an ICU tended to be younger than cases treated in other settings (p=0.043).

The vast majority of fatal influenza cases (97%) were prescribed antiviral drugs. Data regarding the date of antiviral therapy onset were not available. Of 137 cases with available data regarding antibiotic therapy, 136 received antibiotics (median number of antibiotics = 3). More intensive use of antibiotics was observed in patients treated in an ICU (p=0.01). The most common antibiotics administered were meropenem, linezolid, moxifloxacin and vancomycin. 

## **DISCUSSION**

Pandemic influenza A (H1N1) was considerably less lethal than originally expected. The estimation of CFR using the laboratory confirmed cases in the denominator, results in a rate of 0.82%. This finding is consistent with other studies that have used laboratory confirmed cases as a denominator [6] [7]. This approach provides an underestimation of the incidence of all H1N1 cases and therefore an overestimation of the CFR. A lower case fatality ratio is in agreement with data from the EuroMoMo surveillance system operating in Greece, where no excess all-cause mortality was observed until the end of May 2010 [4].

The estimated population mortality rate of 13.23 deaths per million is also subject to uncertainty due to the number of cases that may have died from H1N1 and have remained undiagnosed. Furthermore, the proportion of undiagnosed cases may vary across different countries.

Regarding the time distribution of fatal cases, it follows an epidemic curve with a peak two weeks later than the peak of the sentinel surveillance system. This finding is consistent with the average time from onset of symptoms to death (16 days).

The age distribution of the fatal cases is significantly different from that of the laboratory confirmed cases (Figure 4). As already mentioned, the median age of all fatal cases was 53 years, in contrast to the median age of laboratory confirmed cases (23 years). The elderly appear to be protected from infection to some extent, but when infected, they are more likely to have a fatal outcome than younger cases.

**Figure:**
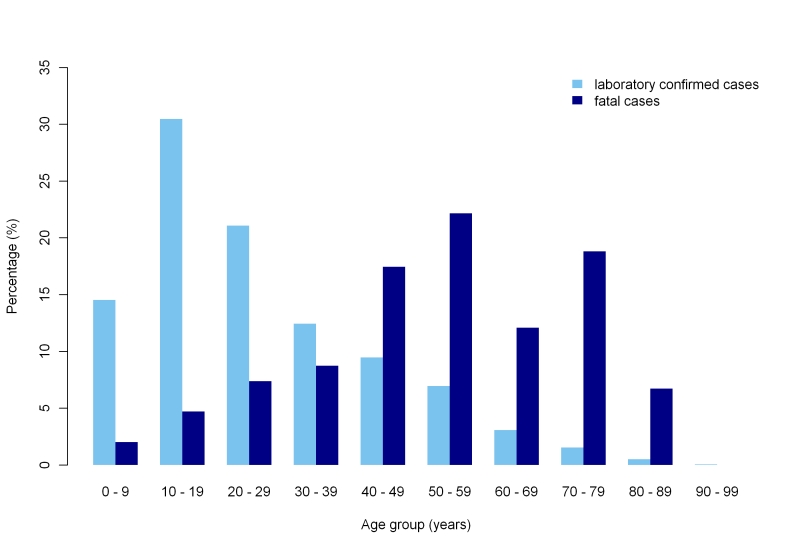

**Figure 4. Age distribution of pandemic influenza A (H1N1) fatal cases and laboratory confirmed cases**

The majority of those who died had at least one underlying disease. However, for a considerable minority of cases who died (20%) no underlying condition was reported. Our findings are in agreement with those reported earlier in the UK, the USA and countries from the southern hemisphere [8] [9] [10]. Pregnant women seem to be under-represented here compared to the results of other studies worldwide [6] [11] [12]. However, it is precarious to draw conclusions regarding pregnancy as a risk factor due to the relatively small number of fatal cases analyzed.

Obesity prevalence was higher among adults in our study (26%), compared to the general adult population obesity prevalence in Greece (17.5%) [13]. Other studies have also identified obesity as one of the most noticeable risk factors among critically ill patients [11] [14] [15]. The impact of obesity was particularly high in the age group 40–59 years (40.6%). Its role, however, remains to be further investigated.

A large proportion of children and teenagers (63%) in our study had neurocognitive or neuromuscular disorders. This is consistent with the findings of a study in the USA [16], in which 61% of children who died from pandemic H1N1 influenza suffered from neurodevelopmental disorders.

Data regarding the time course of illness are comparable with those reported in other studies [8] [11] [15] [16].

A first limitation of this study may be underdiagnosis of fatal cases from pandemic influenza H1N1. The difficulty in determining whether the death is attributable to H1N1 infection or to associated factors remains a major limitation too. A third limitation is the unavailability of data on the time when antiviral therapy started. Last, data on underlying conditions and risk factors are missing for 6% of pandemic fatal cases.

These findings should be taken into consideration when vaccination strategies are employed. Given the low vaccination coverage of the population of Greece [17] [18], persons at high risk for influenza complications should still be given priority. Regarding the vaccination of the whole population, apart from the fact that a substantial minority of deaths occurred among healthy people, the results of cost benefit analyses should also be taken into account. 

## **FUNDING INFORMATION**

This work was funded by the Hellenic Centre for Diseases Control and Prevention (HCDCP) as part of its  surveillance mission; HCDCP is funded by the Hellenic Ministry of Health and Social Solidarity. 

## **COMPETING INTERESTS**

The authors have declared that no competing interests exist.
